# Weight Cycling Enhances Adipose Tissue Inflammatory Responses in Male Mice

**DOI:** 10.1371/journal.pone.0039837

**Published:** 2012-07-25

**Authors:** Sandra Barbosa-da-Silva, Julio C. Fraulob-Aquino, Jessica R. Lopes, Carlos A. Mandarim-de-Lacerda, Marcia B. Aguila

**Affiliations:** Laboratory of Morphometry, Metabolism and Cardiovascular Disease, Biomedical Center, Institute of Biology, State University of Rio de Janeiro, Rio de Janeiro, Brazil; University of Tor Vergata, Italy

## Abstract

**Background:**

Obesity is associated with low-grade chronic inflammation attributed to dysregulated production, release of cytokines and adipokines and to dysregulated glucose-insulin homeostasis and dyslipidemia. Nutritional interventions such as dieting are often accompanied by repeated bouts of weight loss and regain, a phenomenon known as weight cycling (WC).

**Methods:**

In this work we studied the effects of WC on the feed efficiency, blood lipids, carbohydrate metabolism, adiposity and inflammatory markers in C57BL/6 male mice that WC two or three consecutive times by alternation of a high-fat (HF) diet with standard chow (SC).

**Results:**

The body mass (BM) grew up in each cycle of HF feeding, and decreased after each cycle of SC feeding. The alterations observed in the animals feeding HF diet in the oral glucose tolerance test, in blood lipids, and in serum and adipose tissue expression of adipokines were not recuperated after WC. Moreover, the longer the HF feeding was (two, four and six months), more severe the adiposity was. After three consecutive WC, less marked was the BM reduction during SC feeding, while more severe was the BM increase during HF feeding.

**Conclusion:**

In conclusion, the results of the present study showed that both the HF diet and WC are relevant to BM evolution and fat pad remodeling in mice, with repercussion in blood lipids, homeostasis of glucose-insulin and adipokine levels. The simple reduction of the BM during a WC is not able to recover the high levels of adipokines in the serum and adipose tissue as well as the pro-inflammatory cytokines enhanced during a cycle of HF diet. These findings are significant because a milieu with altered adipokines in association with WC potentially aggravates the chronic inflammation attributed to dysregulated production and release of adipokines in mice.

## Introduction

Obesity is associated with low-grade chronic inflammation attributed to the dysregulated production and release of cytokines and adipokines, including tumor necrosis factor (TNF), interleukin (IL)-6, monocyte chemoattractant protein (MCP)-1, leptin, resistin and adiponectin in macrophage-infiltrated abdominal adipose tissue [1]. Infiltration of macrophages into adipose tissue, increased production of pro-inflammatory mediators by adipocytes, and systemic increase of inflammatory cytokines are all associated with obesity [2]. This increase in inflammation may play a role in the pathogenesis of insulin resistance and cardiovascular disease and an increased risk of cardiovascular events is associated with obesity [3]. This is particularly relevant considering that the prevalence of obesity continues to rise and has become a public health problem worldwide [4].

The control of obesity has an impact on cardiovascular disease and other diseases that are commonly associated with an elevated body mass index [5]. Therefore, different therapeutic strategies have been attempted to decrease body adiposity. Nutritional interventions such as dieting, however, are often accompanied by repeated bouts of weight loss and regain, a phenomenon known as weight cycling (WC) [6]. WC occurs frequently in overweight and obese people in their attempts to lose body mass (BM) and to maintain lower BM allowing the speculation that WC may further increase the elevated disease risk that is common with weight gain because WC causes a more profound change in chronic inflammation than sustained weight gain [7]. Moreover, WC has clinical importance related with studies suggesting increased risks of morbidity and mortality in association with fluctuations in BM [8], [9].

The present study aimed to investigate the effects of WC on feed efficiency, blood lipids, glucose-insulin homeostasis and adiposity and adipose tissue inflammatory responses in mice that cycled two or three consecutive times because of a HF diet followed by a standard chow diet, and a standard chow diet followed by a HF diet.

## Methods

Animal protocol was approved by the Animal Ethics Committee of the State University of Rio de Janeiro (Protocol Number CEA/003/2009), and the procedures were in accordance with the guidelines for experimentation with animals (NIH Publication N°. 85-23, revised 1996). Male C57BL/6 mice (3-mo-old) were housed under a controlled temperature (21±1°C), humidity (60±10%) and 12 h light/dark cycle (1:00 AM to 01:00 PM light).

### Experimental diets

The diets were manufactured in accordance with the AIN-93M recommendations [10]. The standard chow (SC) had 76% energy from carbohydrates, 14% energy from protein, and 10% energy from lipids (40 g soybean oil/Kg diet) (total energy 15 kJ/g). The high-fat diet (HF) had 26% energy from carbohydrates, 14% energy from protein, 50% energy from animal lard (320 g/Kg diet) and 10% energy from lipids (40 g soybean oil/Kg diet) (total energy 21 kJ/g). The mineral and vitamin contents were identical in both diets and mice had free access to food and water during the experimental period.

### Experimental design (Figure 1)

**Figure 1:**
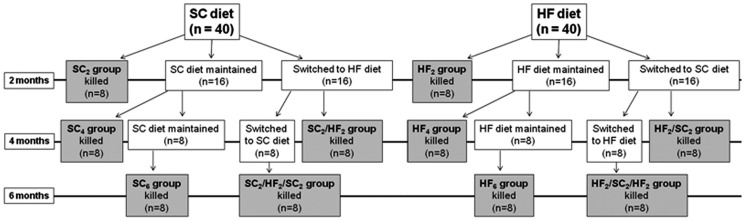
Schematic design of the experiment detailing the formation of the groups and sample size. SC is standard chow; HF is high-fat diet; n is the number of mice in the sample. The subscript number after the acronym of the group indicates the number of months the diet was consumed by animals. The gray charts represent the final groups studied.

Eighty mice were randomly allocated into 10 groups (n = 8/group) and studied for two, four or six months according to the details showed in the Figure 1. In summary, 40 mice received SC for two months (SC2 group, non-WC mice), at which time eight mice were killed. The remaining 32 were divided into the groups: b) 16 mice remained on SC diet for more two months (SC4 group, non-WC mice); c) 16 mice were switched to the HF diet for two months (SC2/HF2 group). At which time, eight mice of SC4 group were killed and eight mice of SC2/HF2 group were killed. The remaining 16 mice were divided into the groups: d) 8 mice remained on SC diet for more two months (SC6 group, non-WC mice), e) 8 mice were switched to the SC diet for two months (SC2/HF2/SC2 group), at which time all animals were killed. The same schema was used for HF diet: f) 40 mice received HF diet for two months (HF2 group, non-WC mice), at which time eight mice were killed. The remaining 32 mice were divided into the groups: g) 16 mice remained on HF diet for more two months (HF4 group, non-WC mice); h) 16 were switched to the SC diet for two months (HF2/SC2 group). At which time, eight mice of HF4 group were killed and eight mice of HF2/SC2 group were killed. The remaining 16 were divided into following groups: i) 8 mice remained on HF diet for more two months (HF6 group, non-WC mice), j) 8 were switched to the HF diet for two months (HF2/SC2/HF2 group), at which time all remained animals were killed.

### Body Mass, Food Intake and Feed Efficiency

Mice had free access to food and water during the experimental period, and their intake was monitored daily. In addition, their body mass (BM) was measured each week. The BM was measured 2:00 PM every Thursday (i.e., the middle of the week during the dark cycle). The start of the week to purpose of weight-cycling was considered Monday 07:00 AM. Therefore, a complete week started and finished on Monday 07:00 AM, seven days apart.

Fresh chow was provided daily, and any remaining chow from the previous day was discarded. Food consumption was determined as the difference between the food supplied and the amount of food left in the grid. Energy intake was the product of food consumption by the energy content of the diet. The energy intake per gram of body mass gained, termed the feed efficiency (FE), was calculated as a digestive and metabolic indicator of the ease that energy consumed was added as body mass. We calculated the FE as the ratio between the BM gain in grams and the food consumed in kJ per animal presented as percentage.

### Blood Glucose Test Analysis

A blood glucose test, the oral glucose tolerance test (OGTT) was conducted prior to the initiation of diet feeding and after each cycle of HF and SC feeding. For blood glucose levels, blood was obtained by milking the tail after a small incision was made to the animal's tail (glucometer Accu-chek, Roche Diagnostic, Manheim, Germany). The OGTT was performed using a 25% solution of glucose in sterile saline (0.9% NaCl) at a dose of 1.0 g/kg. The glucose was administered by orogastric gavage after a six-hour fasting period. The blood glucose concentration was measured before glucose administration (0 min) and 15, 30, 60 and 120 minutes after administration.

### Euthanasia

On the day before euthanasia, animals were deprived of food for 6 hours prior to being anesthetized (intraperitoneal sodium pentobarbital, 150 mg/kg) and then blood samples were obtained by cardiac puncture through the right atrium. The serum was obtained after blood centrifugation at room temperature (120× g for 15 min).

Total cholesterol (TC) and triglycerides (TG) were assayed by an enzymatic colorimetric method according to manufacturer instructions. Biochemical analyses were performed in a semi-automatic spectrophotometer (Bioclin, Belo Horizonte, MG, Brazil).

The inguinal fat pad located between the lower part of the rib cage and the mid-thigh was considered subcutaneous fat. The retroperitoneal fat (connected to the posterior abdominal wall near the kidneys and the abdominal portion of the ureters) and the epididymal fat (located in the lower part of the abdomen and connected to the epididymis) were considered intra-abdominal fat pad. Therefore, after the animals were euthanized, the subcutaneous and intra-abdominal fat pads were carefully dissected and weighed. The adiposity index was determined as the ratio between the sum of the masses intra-abdominal and subcutaneous divided by the total body mass, presented as a percentage.

### Milliplex Map Immunoassay

Serum insulin, adiponectin, leptin, resistin, IL-6, TNF-alpha, and MCP-1 levels were measured using Multiplex Biomarker Immunoassays for Luminex xMAP technology (Millipore, Billerica, MA, USA, cat. no. MADPK7107-M). The intra-essay and inter-assay precision of the mouse serum adipokine panel were <6% and <11%, respectively.

### Adipocyte quantification

Epididymal adipose tissue samples were fixed in freshly prepared formaldehyde in 4% w/v 0.1 M phosphate buffer pH 7.2 for 48 h and then embedded in Paraplast plus (Sigma-Aldrich, St. Louis, MO, USA), sectioned at 5 µm and stained with hematoxylin and eosin. Ten non-consecutive microscopic fields were analyzed per animal on a light microscope (Olympus BX51, Olympus America Inc., Miami, FL, USA) and digital images were taken (TIFF format, 36-bit color, 1280×1024 pixels). At least 50 adipocytes per animal had their mean diameter measured using the Image Pro plus software v7.01 (Media Cybernetics, Silver Spring, MD, USA). In addition, the numerical density per area of adipocytes was evaluated considering the number of adipocytes into a frame of known area produced with the STEPanizer web-based system (www.stepanizer.com) [11]. The adipocytes were counted into the frame when they did not hit the “forbidden lines” or its extensions [12].

### Western Blotting

The total protein content from the intra-abdominal fat pad was extracted in homogenizing buffer containing protease inhibitors. Homogenates were centrifuged twice for 10 minutes at 4°C, infranatants were collected, and protein concentration was then determined using the BCA protein assay kit (Thermo Scientific, Rockford, IL, USA). After denaturation, proteins were separated by electrophoresis on a polyacrylamide gel (SDS-PAGE) and transferred to a nitrocellulose membrane. The adiponectin (A6354, Sigma Aldrich), leptin (L3410, Sigma Aldrich), and IL-6 (AB1423 Millipore) proteins were studied. Immunoreactive bands were detected with the ECL reagent kit (GE Healthcare BioSciences, Buckinghamshire, UK), and signals were visualized by autoradiography and quantified using the Imagepro Plus. The expression of the structural anti-beta actin was used to correct the blot data (Mouse, Monoclonal – Santa Cruz, Biotechnology).

### Statistical Analysis

A regression analysis plotted BM (as dependent variable, *y*) against the weeks of the experiment (as independent variable, *x*). This bivariate study used log-transformed data and the allometric model *log y = log a+b log x* establishing the BM growth rates [13]. Linear regressions made for each specific cycle of diet were compared using *t*-test (comparison of slopes test). The Pearson's coefficient of correlation was used to determine the significance of each regression. In addition, data were tested for normality and homogeneity of variances and the differences among groups were tested, when appropriated, with one-way ANOVA nad post-hoc test of Bonferroni, one-way ANOVA with repeated measures and post-hoc test of Bonferroni, or unpaired *t*-test. A *P*-value<0.05 was considered statistically significant (Prism v. 5.04 for Windows, GraphPad Software, San Diego, CA, USA).

## Results

Data are show as mean and standard error of the mean.

### Body mass (Tables 1 and 2, Figure 2)

**Figure 2:**
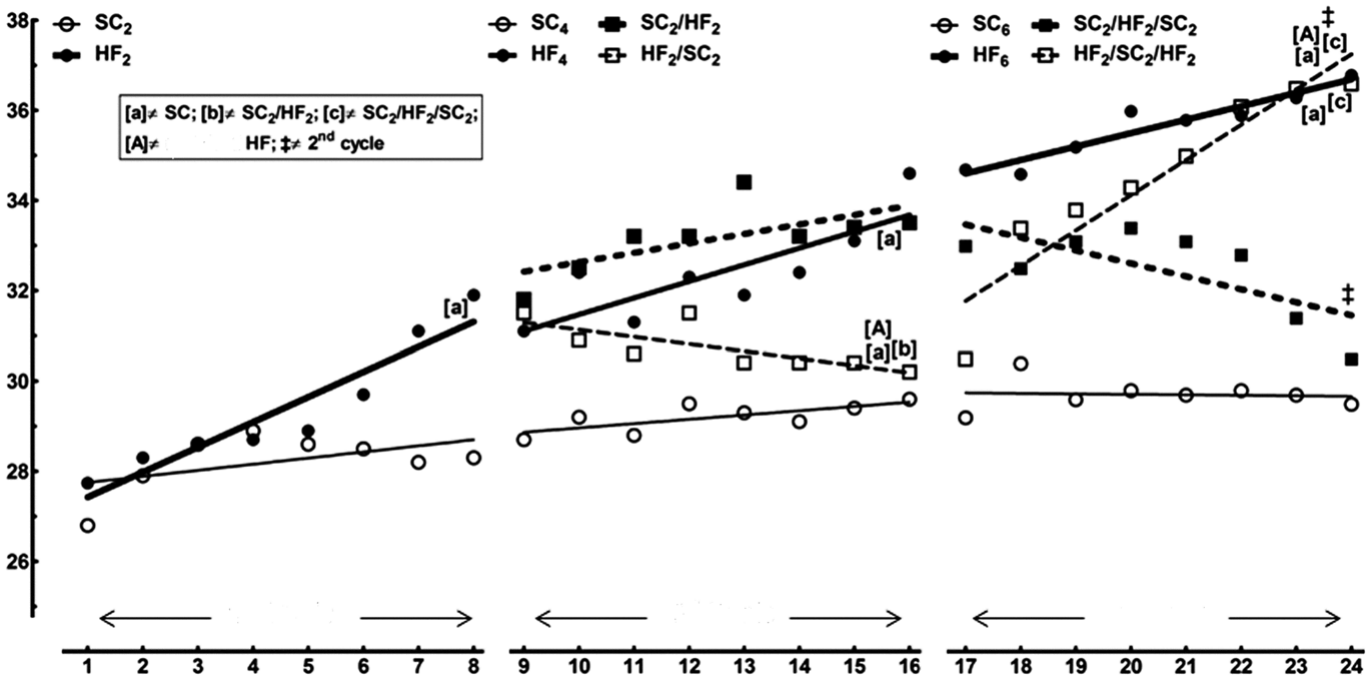
Trends of the body mass evolution determined using the allometric model *log y = a+b log x*. The regressions show how the standard chow (SC) or the high-fat diet (HF), or the switching between these two diets throughout the experiment changed the body mass of the animals. In the signaled cases (comparison of slopes), *P*<0.05, when: [a] different from SC; [b] different from SC2/HF2; [c] different from SC2/HF2/SC2; [A] different from HF counterpart; § different from 2^nd^ cycle. The details about the calculated equations as well as the significance of the comparison of their slopes are shown in Table 2.

**Table 1:** Body Mass (BM) (in gram, values are means with their standard error) and percentage BM gain in experimental groups after 1^st^, 2^nd^ and 3^th^ cycles, where SC is standard chow and HF is the high-fat diet.

Groups	1st Cycle	2nd Cycle	3rd Cycle
	(weeks 1–8)	(weeks 9–16)	(weeks 17–24)
SC_2_	28.3±0.8		
HF_2_	33.3±0.9[a]		
SC_4_		29.4±0.6	
HF_4_		34.6±1.7[a]	
SC_2_/HF_2_		33.6±0.9[a, †]	
HF_2_/SC_2_		30.0±0.6[b, ‡]	
SC_6_			29.5±0.4
HF_6_			36.8±1.0[a]
SC_2_/HF_2_/SC_2_			30.4±0.3[b, Δ]
HF_2_/SC_2_/HF_2_			36.6±1.0[a, c, §]

Differences among groups were analyzed using one-way ANOVA and post hoc test of Bonferroni (in the signaled cases, *P*<0.05, when:

[a] different from SC counterpart;

[b] different from HF counterpart,

[c] different from SC_2_/HF_2_/SC_2_).

Differences among the cycles were analyzed using one-way ANOVA of repeated measures and post-hoc test of Bonferroni (in the signaled cases, *P*<0.05, when:

[†] different from SC_2_;

[‡] different from HF_2_,

[Δ] different from SC_2_/HF_2_,

[§] different from HF_2_/SC_2_.

**Table 2:** Linear regressions of the body mass against the cycles of different diets (in weeks) based on the allometric model with log-transformed data, log y = log a+b log x (where a is the intercept and b is the slope).

Variable X (weeks)	Variable Y (body mass)	Intercept	Slope	*R*	*P*
1–8	SC_2_	1.436	0.024	0.747	0.03
	HF_2_	1.429	0.086	0.914	0.002
9–16	SC_4_	1.421	0.041	0.753	0.03
	HF_4_	1.364	0.134	0.796	0.02
	SC_2_/HF_2_	1.430	0.083	0.724	0.04
	HF_2_/SC_2_	1.560	−0.066	−0.776	0.02
17–24	SC_6_	1.488	−0.011	−0.118	Ns.
	HF_6_	1.330	0.169	0.940	<0.0001
	SC_2_/HF_2_/SC_2_	1.747	−0.180	−0.679	Ns.
	HF_2_/SC_2_/HF_2_	0.917	0.474	0.945	<0.0001

Comparison of Slopes		*t*-test	*P*		
SC_2_	HF_2_	3.423	0.005		
SC_4_	HF_4_	2.108	0.05		
SC_6_	HF_6_	3.883	0.002		
SC_2_	SC_4_	0.657	Ns.		
SC_4_	SC_6_	1.376	Ns.		
SC_4_	SC_2_/HF_2_	1.189	Ns.		
SC_4_	HF_2_/SC_2_	4.060	0.002		
SC_6_	SC_2_/HF_2_/SC_2_	1.902	Ns.		
SC_6_	HF_2_/SC_2_/HF_2_	6.239	<0.0001		
SC_2_/HF_2_	SC_2_/HF_2_/SC_2_	3.323	0.006		
SC_2_	SC_2_/HF_2_	1.783	Ns.		
HF_2_	HF_2_/SC_2_	3.504	0.004		
HF_2_	HF_4_	0.951	Ns.		
HF_4_	HF_6_	0.581	Ns.		
HF_4_	HF_2_/SC_2_	4.254	0.001		
HF_4_	SC_2_/HF_2_	0.963	Ns.		
HF_6_	HF_2_/SC_2_/HF_2_	4.249	0.001		
HF_6_	SC_2_/HF_2_/SC_2_	4.189	0.001		
HF_2_/SC_2_	HF_2_/SC_2_/HF_2_	8.562	<0.0001		
SC_2_/HF_2_	HF_2_/SC_2_	3.819	0.002		
SC_2_/HF_2_/SC_2_	HF_2_/SC_2_/HF_2_	6.281	<0.0001		

*R* is the coefficient of correlation of Pearson, *P* is the probability. The group abbreviations were defined in the text (Material and Methods). Ns, not significant.

The animals started the study with no significant difference in their initial BM. Table 1 shows the BM evolution throughout the experiment. After two months of diet, the HF2 mice had greater BM than SC2 mice (+18%, *t*-test, *P<*0.001). At four months of diet (second cycle of diet), the HF4 mice had greater BM than SC4 mice (+17%, one-way ANOVA and post-hoc test of Bonferroni, *P<*0.0001) and the SC2/HF2 mice had greater BM than both SC2 and SC4 mice (+19% and +14%, one-way ANOVA of repeated measures and post-hoc test of Bonferroni, *P<*0.001). However, the HF2/SC2 mice showed smaller BM than both HF2 and HF4 mice (−10% and −13%, one-way ANOVA of repeated measures and post-hoc test of Bonferroni, *P<*0.001). At six months of diet (third cycle of diet), both HF6 and HF2/SC2/HF2 mice were 25% heavier than SC6 mice (one-way ANOVA and post-hoc test of Bonferroni, *P<*0.001). The HF2/SC2/HF2 mice were also 20% heavier than SC2/HF2/SC2 mice (one-way ANOVA and post-hoc test of Bonferroni, *P<*0.001) and 22% heavier than HF2/SC2 mice (one-way ANOVA of repeated measures and post-hoc test of Bonferroni, *P<*0.001). In addition, the SC2/HF2/SC2 mice were lighter than HF6 mice (−9.5%, one-way ANOVA and post-hoc test of Bonferroni, *P<*0.01) and lighter than SC2/HF2 mice (−17%, one-way ANOVA of repeated measures and post-hoc test of Bonferroni, *P<*0.001).

The BM growth rates (slopes determined by the allometric method) in each specific cycle of diet are detailed in Table 2. All regressions were statistically significant, except for SC6 and SC2/HF2/SC2 groups. In the first cycle of diet, the BM growth rate was higher for HF2 than for SC2. In the second cycle of diet, the HF4 group had the higher BM growth rate, followed in the sequence by the SC2/HF2 group and the SC4 group. The HF2/SC2 group showed negative BM growth rate. In the third cycle of diet, the regressions were not significant for the groups SC6 and SC2/HF2/SC2. The BM growth rate was greater to the HF2/SC2/HF2 group than to the HF6 group.

The changes observed in the growth rates of the BM throughout the experiment are showed in Figure 2. The first cycle has only two groups, SC2 and HF2. These groups have very different trends of fat that remain different in the following two cycles of diet (SC4 and SC6, HF4 and HF6). In the second cycle of diet, replacing the HF diet for SC (HF2/SC2 group) caused an important alteration in the tendency to gain weight. Rather, switching the SC to the HF diet (group SC2/HF2) put this group closer to the HF4 group. There is no difference between the growth rates of the BM between the groups SC4 and SC2/HF2 because their tendencies in BM gain are practically parallel, although there was a greater BM in the SC2/HF2 group. In the third cycle of diet, the animals are still more separated by their BM. There are inflexions in the growth rates of the BM for the groups SC2/HF2/SC2 and HF2/SC2/HF2 in comparison with the previous cycle. At the end of this cycle, the HF2/SC2/HF2 group had the higher growth rate, different from virtually all the other groups of this cycle, including their own growth rate from the previous cycle.

### Food intake and feed efficiency (Table 3)

**Table 3:** Food intake, feed efficiency, blood total cholesterol, triglycerides, oral glucose tolerance test (OGTT, a.u.c is the area under de curve in arbitrary units), serum insulin of groups after the cycles of diets.

Parameters			Groups	
1^st^ cycle (2 months)	SC_2_			HF_2_
Food intake (g/animal/day)	3.72±0.09			3.20±0.05
Feed Efficiency (kJ) ×100	0.47±0.03			1.25±0.09[a]
Total Cholesterol (mg/dl)	81.5±3.3			129.0±5.6[a]
Triglycerides (mg/dl)	34.8±3.4			50.8±1.1[a]
OGTT (a.u.c.)	1005.0±9.1			1244.0±14.1[a]
Insulin (µIU/L)	36.2±1.8			59.2±3.9[a]

2^nd^ cycle (4 months)	SC_4_	HF_4_	SC_2_/HF_2_	HF_2_/SC_2_
Food intake (g/animal/day)	3.70±0.03	3.10±0.10	3.30±0.04	3.70±0.06
Feed Efficiency (kJ) ×100	0.62±0.01	1.53±0.09[a]	1.64±0.08[a]	0.80±0.04[b,c]
Total Cholesterol (mg/dl)	128.2±3.6	211.0±12.9[a]	179.4±7.5[a]	127.2±2.2[b,c]
Triglycerides (mg/dl)	28.8±3.2	52.8±1.8[a]	52.4±3.2[a]	40.3±3.1[b,c]
OGTT (a.u.c)	1076±13	1238±15[a]	1283±23[a]	1254±17[a]
Insulin (µIU/L)	34.8±3.0	53.4±0.2[a]	47.5±3.2[a]	48.6±2.5[a]

3^rd^cycle (6 months)	SC_6_	HF_6_	SC_2_/HF_2_/SC_2_	HF_2_/SC_2_/HF_2_
Food intake (g/animal/day)	3.40±0.05	3.20±0.08	3.30±0.09	3.13±0.05
Feed Efficiency (kJ) ×100	0.23±0.04	0.67±0.04[a]	0.30±0.04[b]	0.68±0.06[a,d]
Total Cholesterol (mg/dl)	111.4±2.8	176.3±12.0[a]	112.5±7.6[b]	163.6±4.3[a,d]
Triglycerides (mg/dl)	37.0±2.0	67.8±2.2[a]	39.2±3.4[b]	78.0±6.5[a,d]
OGTT (a.u.c)	1010±18	1331±28[a]	1148±8[a]	1283±23[a,d]
Insulin (µIU/L)	26.8±1.7	45.6±1.9[a]	34.0±0.5[a,b]	39.1±2.3[a]

SC is standard chow; HF is high-fat diet; n is the number of mice in the sample. The subscript number after the acronym of the group indicates the number of months the diet was consumed by animals. Values are means and standard error of the mean (n = 8 per group In the signaled cases (one-way ANOVA and post-hoc test of Bonferroni), *P<*0.05, when:

[a] different from SC counterpart;

[b] different from HF counterpart,

[c] different from SC_2_/HF_2_;

[d] different from SC_2_/HF_2_/SC_2_.

Mice fed SC and HF diets had similar food intake (per gram of diet) throughout the entire experiment. However, the FE was much inferior in the SC groups (SC2, SC4 and SC6) compared with their HF counterparts.

At 4 months: The FE of animals was higher during HF diet feeding and smaller during SC feeding. The FE of SC2/HF2 group significantly was higher than SC4 (+164%) and was not different from HF4. In addition, after the switch to SC, the FE of the HF2/SC2 group significantly decreased in comparison with the HF4 group (−48%) and was not different from the SC4 group. In addition, we found a significant difference in the FE between the groups SC2/HF2 and HF2/SC2 (−51%).

At 6 months: After three consecutive cycles, mice FE decreased with SC feeding and increased with HF feeding. The FE of the SC2/HF2/SC2 group was not different in comparison with SC6, but it was different from HF6 (−55%). The FE of HF2/SC2/HF2 significantly increased in comparison with SC6 (+196%) and was not different from the value found in the HF6 group. In addition, we found a significant difference in the FE between the groups SC2/HF2/SC2 and HF2/SC2/HF2 (+127%).

Comparing the same group at different ages, there was no difference in food intake and FE among the SC2, SC4 and SC6 groups and among the HF2, HF4 and HF6 groups.

### Biochemistry

#### *a)* Serum Total Cholesterol (Table 3)

In HF2, HF4 and HF6, the serum total cholesterol (TC) was significantly higher in comparison with TC of SC counterparts.

At 4 months: After 2-months switch to HF feeding, the TC of SC2/HF2 group significantly increased in comparison with SC4 group (+40%) and was not different of the value found in the HF4 group. In addition, after switching to SC feeding, the TC of HF2/SC2 group significantly decreased in comparison with HF4 group (−40%) and SC2/HF2 group (−29%).

At 6 months: The TC decreasing during SC feeding and then rose to a significantly higher levels during HF feeding. The TC of SC2/HF2/SC2 group was not different in comparison with the SC6 group, but it was smaller than the value found in the HF6 group (−36%). In addition, the TC of HF2/SC2/HF2 group significantly increased in comparison with SC6 group (+47%) and with SC2/HF2/SC2 group (+45%).

#### *b)* Serum Triglycerides (Table 3)

In HF2, HF4 and HF6, the serum total triglycerides (TG) were significantly higher in comparison with TG of SC counterparts.

At 4 months: After 2-months switch to HF feeding, the TG of SC2/HF2 group significantly increased in comparison with SC4 group (+82%) and was not different of the value found in the HF4 group. In addition, after switching to SC feeding, the TG of HF2/SC2 group significantly decreased in comparison with HF4 group (−24%) and SC2/HF2 group (−23%).

At 6 months: Interesting, after three weight cycles, the TG decreasing during SC feeding and then rose to a significantly higher levels during HF feeding. The TG of SC2/HF2/SC2 group was not different in comparison with the SC6 group, but it was smaller than the value found in the HF6 group (−42%). In addition, the TG of HF2/SC2/HF2 group significantly increased in comparison with SC6 group (+111%) and in comparison with SC2/HF2/SC2 group (+99%).

### Oral Glucose Tolerance Test – OGTT (Table 3)

At beginning of the experiment, at 3 months of age, all mice studied displayed normal glucose tolerance (data not showed). However, in HF2, HF4 and HF6 groups, the OGTT were significantly higher in comparison with OGTT of SC counterparts.

At 4 months: The OGTT was markedly increased in the HF4 and SC2/HF2 groups, and the OGTT did not fully normalized to the level of the SC4 group in the HF2/SC2 group after switching to SC feeding.

At 6 months: After the three consecutive cycles, the OGTT levels increasing during HF feeding, as was seen in the HF6, SC2/HF2/SC2 and HF2/SC2/HF2 groups and then the values did not return to normalize levels during SC feeding. In addition, the OGTT levels of HF2/SC2/HF2 group significantly increased in comparison with SC2/HF2/SC2 group (+12%).

### Serum Insulin (Table 3)

In HF2, HF4 and HF6, the insulin levels were significantly higher in comparison with serum insulin of SC counterparts.

At 4 months: The insulin levels were markedly increased in the HF4 and SC2/HF2 groups, and the insulin levels did not fully normalized to the level of the SC4 group in the HF2/SC2 group after switching to SC feeding.

At 6 months: After the three consecutive cycles, the insulin levels increased during HF feeding, as was seen in the HF6, SC2/HF2/SC2 and HF2/SC2/HF2 groups and then the values did not return to normalize levels during SC feeding. In addition, the insulin levels of SC2/HF2/SC2 group significantly decreased in comparison with HF6 group (−25%).

### Adipose tissue

#### *a)* Fat Pad Mass (Table 4)

**Table 4:** Fat pad masses and serum adipokines in the cycles of the diet.

1^st^ cycle (2 months)			SC_2_	HF_2_
Intra-abdominal fat pad (g)			0.17±0.02	1.6±0.09[a]
Subcutaneous fat pad (g)			0.12±0.02	0.83±0.04[a]
Adiposity index (%)			1.02±0.12	7.73±0.5[a]
Q_A_[adipocytes]/mm^2^			33.0±1.0	14.0±0.7[a]
Adiponectin (10^5^ pg/mL)			100±13	60±1[a]
Leptin (pg/mL)			366.5±99.8	9183.9±914.4[a]
Resistin (pg/mL)			4358.2±354.7	5424.9±687.0[a]
IL-6 (pg/mL)			34.7±8.2	126.5±18.7[a]
TNF-alpha (pg/mL)			3.3±0.2	5.0±0.5[a]
MCP-1 (pg/mL)			27.7±6.8	31.5±4.3

2^nd^ cycle (4 months)	SC_4_	HF_4_	SC_2_/HF_2_	HF_2_/SC_2_
Intra-abdominal fat pad (g)	0.13±0.001	1.91±0.08[a]	1.20±0.22[a,b]	0.41±0.03[b, c]
Subcutaneous fat pad (g)	0.11±0.01	1.40±0.14[a]	0.55±0.09[a, b]	0.27±0.04[b]
Adiposity index (%)	0.80±0.08	9.67±0.43[a]	5.98±0.97[a,b]	2.24±0.11[b,c]
Q_A_[adipocytes]/mm^2^	34.0±1.5	16.0±0.6[a]	17.0±1.4[a]	30.0±0.5[b,c]
Adiponectin (10^5^ pg/mL)	110±5	80±1[a]	60±5[a]	100±4[b, c]
Leptin (pg/mL)	331.0±69.0	9268.3±1495.2[a]	12393.8±1931.0[a]	2079.6±581.9[b, c]
Resistin (pg/mL)	5424.9±687.0	7500.4±240.6[a]	8098.9±437.4[a]	6181.2±368.7[c]
IL-6 (pg/mL)	32.7±8.5	99.1±5.2[a]	93.80±9.6[a]	47.6±3.2[b, c]
TNF-alpha (pg/mL)	3.2±0.2	5.0±0.5[a]	4.8±0.3[a]	3.1±0.2[b, c]
MCP-1 (pg/mL)	12.1±5.9	16.9±9.1	25.5±11.6	20.3±2.1

3^rd^cycle (6 months)	SC_6_	HF_6_	SC_2_/HF_2_/SC_2_	HF_2_/SC_2_/HF_2_
Intra-abdominal fat pad (g)	0.66±0.07	2.02±0.17[a]	0.87±0.21[b]	2.21±0.19[a,d]
Subcutaneous fat pad (g)	0.25±0.03	1.72±0.11[a]	0.39±0.08[b]	0.75±0.09[a,b,d]
Adiposity index (%)	3.10±0.20	10.16±0.35[a]	4.12±0.94[b]	8.09±0.38[a,d]
Q_A_[adipocytes]/mm^2^	32.0±1.4	26.0±0.6	22.0±1.3	16.0±1.5[a,b,d]
Adiponectin (10^5^ pg/mL)	130±4	80±2[a]	110±6[a,b]	80±1[a,d]
Leptin (pg/mL)	1,232.6±298.1	10,381.7±1030.3[a]	1,104.4±234.6[b]	9,965.5±1621.0[a,d]
Resistin (pg/mL)	4,459.5±390.8	6,186.9±368.1[a]	3,791.0±285.1[b]	6,263.7±464.3[a,d]
IL-6 (pg/mL)	21.4±2.4	100.4±7.8[a]	73.6±13.8[a]	93.9±9.2[a]
TNF-alpha (pg/mL)	3.6±0.2	6.1±0.6[a]	4.9±0.3	5.3±0.3[a]
MCP-1(pg/mL)	22.1±7.5	43.8±13.6	23.6±7.7	28.6±14.3

Values are mean and standard error of the mean (n = 8 per group). In the signaled cases (one-way ANOVA and post-hoc test of Bonferroni), *P<* 0.05, when:

[a] different SC counterpart;

[b] different HF counterpart,

[c] different from SC_2_/HF_2_; and,

[d] different from SC_2_/HF_2_/SC_2_. Q_A_[adipocytes] is numerical density per area of adipocytes.

In HF2, HF4 and HF6, the masses of intra-abdominal and subcutaneous fat pads were significantly higher in comparison with intra-abdominal and subcutaneous fat pads of SC counterparts.

At 4 months: Intra-abdominal and subcutaneous fat pads changed similarly during WC in these groups. In SC2/HF2, intra-abdominal (+823%) and subcutaneous (+400%) fat pads were significantly increased in comparison with SC4. In this group, the intra-abdominal and the subcutaneous fat pads were smaller from HF4 group (−37% and −61%, respectively). In addition, after the switch to SC, intra-abdominal (−78%) and subcutaneous (−81%) fat pads of HF2/SC2 significantly decreased in comparison with HF4 and were not different from SC4. A significant difference was found in the intra-abdominal fat pad between SC2/HF2 and HF2/SC2 (−66%).

At 6 months: The mass of the intra-abdominal and subcutaneous fat pads of SC2/HF2/SC2 were not different in comparison with SC6 group, but both fat pads were smaller in comparison with HF6 (−57% to intra-abdominal and −77% to subcutaneous fat pad). Intra-abdominal (+235%) and subcutaneous (+200%) fat pads of HF2/SC2/HF2 significantly increased in comparison with SC6, but only the subcutaneous fat pad was significantly smaller in comparison with HF6 (−56%). In addition, a significant difference was found in both fat pads between SC2/HF2/SC2 and HF2/SC2/HF2 (+154% to intra-abdominal and +92% to subcutaneous fat pads, respectively).

#### *b)* Adiposity index (Table 4)

We have summed the weight of the intra-abdominal and subcutaneous fat pads, multiplied by 100, and divided by the body weight. This adiposity index increased significantly in HF2, HF4 and HF6 groups in comparison with SC counterparts.

At 4 months: The adiposity index changed similarly during WC in these groups. In SC2/HF2, this index increased significantly in comparison with SC4 group (+647%), but it decreased in comparison with HF4 group (−38%). After switching to SC feeding, this index in HF2/SC2 group was similar to SC4 group, but it was smaller in comparison with HF4 group (−77%) and in comparison with SC2/HF2 group (−62%).

At 6 months: After three WCs, adiposity index decreased during SC and increased during HF feeding. In the SC2/HF2/SC2 group, adiposity index was not different from the SC6 group but it decreased in comparison with the HF6 group (−59%). In the HF2/SC2/HF2 group, this index significantly increased in comparison with SC6 (+161%) and was not different from the HF6 group. In addition, the adiposity index increased significantly in HF2/SC2/HF2 (+96%) in comparison with SC2/HF2/SC2 group.

Comparing the same group at different ages, there was significant differences in adiposity index between the SC2 and SC6 groups (+204%) and between SC4 and SC6 groups (+287%). In relation to HF groups, there was statistical difference between HF2 and HF4 groups (+25%) and between HF2 and HF6 groups (+31%).

#### *c)* Adipocyte Morphometry (Figure 3)

**Figure 3:**
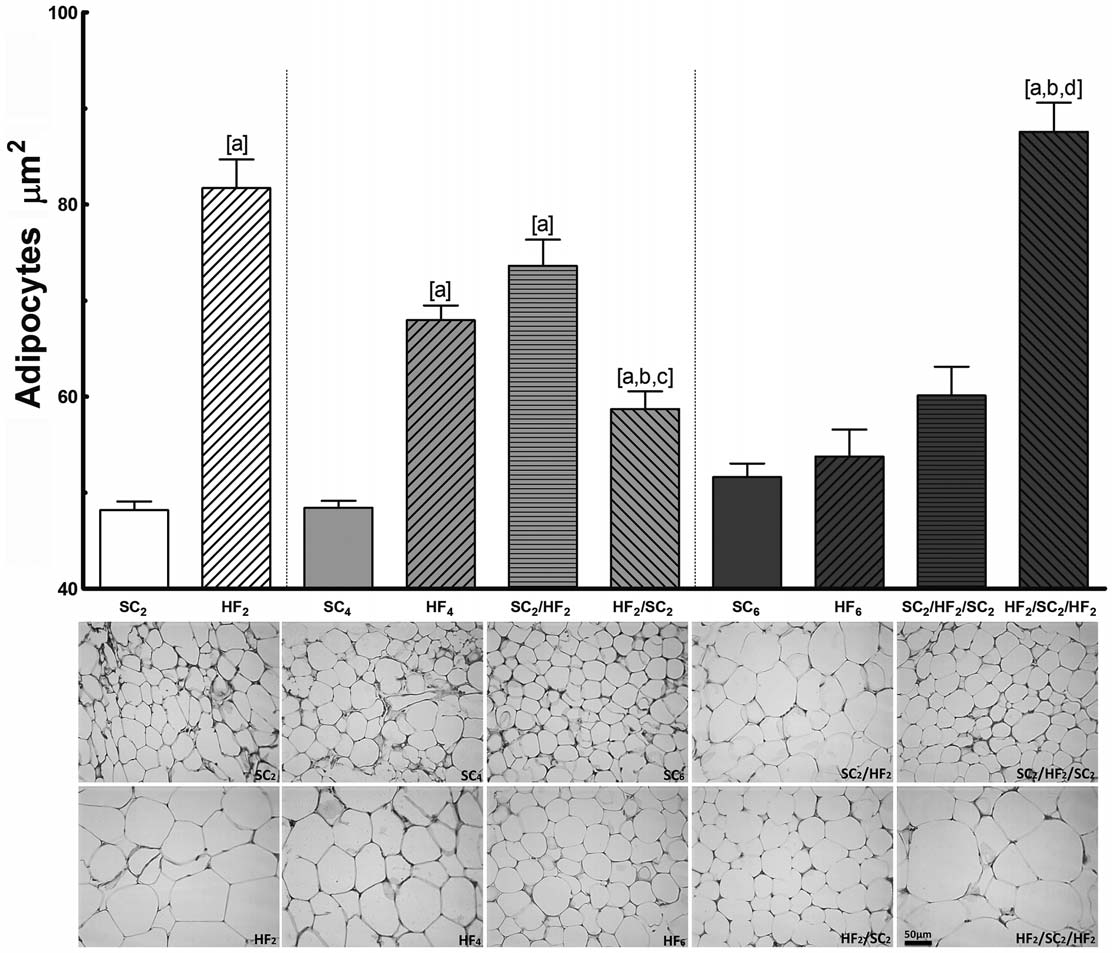
Sectional area of adipocytes and photomicrographs of corresponding groups (n = 8 per group). Values are means with their standard error shown by vertical bars. In the signaled cases (one-way ANOVA and post-hoc test of Bonferroni), *P<*0.05, when: [a] different from SC counterpart; [b] different from HF counterpart; [c] different from SC_2_/HF_2_; and [d] different from SC_2_/HF_2_/SC_2_.

HF2 and HF4 adipocyte sizes were considerably enlarged in comparison with their SC counterparts.

At 4 months: Adipocyte sizes enlarged and decreased concurrently with the change in the fat pad weight. SC2/HF2 had significantly enlarged final adipocyte sizes compared to SC4 (+52%) and was not different from the value found in the HF4 group. In addition, HF2/SC2 had significantly smaller adipocyte sizes in comparison with HF4 (−14%) but was enlarged compared to SC4 (+21%). A significant difference was found in the adipocyte sizes between SC2/HF2 and HF2/SC2; it was 25% larger in SC2/HF2 than in HF2/SC2.

At 6 months: HF6, SC6 and SC2/HF2/SC2 had similar adipocyte sizes. After three WCs, only the adipocyte sizes of HF2/SC2/HF2 were significantly larger than the other groups.

Comparing the same group at different ages, there was no differences in adipocyte sizes among SC groups. In relation to HF groups, there was statistical difference between HF2 and HF4 groups (−16%), between HF2 and HF6 groups (−34%) and between HF4 and HF6 groups (−21%).

#### *d)* Numerical density of adipocytes per area (QA [adipocytes]mm2) (Table 4)

HF2 and HF4 numerical density of adipocytes per area decreased considerably in comparison with their SC counterparts.

At 4 months: The SC2/HF2 group had a significantly smaller numerical density of adipocytes per area compared to SC4 (−29%) and was not different from the value found in the HF4 group. In addition, HF2/SC2 had significantly bigger numerical density of adipocytes per area in comparison with HF4 (+87%) but was not different from the value in the SC4 group. A significant difference was found in the numerical density of adipocytes per area between SC2/HF2 and HF2/SC2; it was 76% bigger in HF2/SC2 than in SC2/HF2 group.

At 6 months: HF6, SC6 and SC2/HF2/SC2 groups had similar numerical density of adipocytes per area. After three WCs, only the numerical density of adipocytes per area of HF2/SC2/HF2 group was significantly bigger than the other groups.

Comparing the same group at different ages, there was no differences in numerical density of adipocytes per area among SC groups. In relation to HF groups, there was statistical difference between HF2 and HF6 groups (+86%), and between HF4 and HF6 groups (+62%).

### Adipokines (Table 4)

#### *a)* Serum Adiponectin

HF2, HF4 and HF6 adiponectin was significantly lower in comparison with the SC counterparts.

At 4 months: Adiponectin was lower during HF and higher during SC feeding. SC2/HF2 adiponectin significantly decreased in comparison with SC4 (−40%) and was not different from HF4. In addition, after the switch to SC, the adiponectin levels of HF2/SC2 significantly increased in comparison with HF4 (+25%) and SC2/HF2 (+67%).

At 6 months: HF6 adiponectin was significantly lower than SC6 (−38%). After three WC, adiponectin increased during SC and decreased during HF feeding. SC2/HF2/SC2 adiponectin was lower in comparison with SC6 (−15%) and was higher in comparison with HF6 (+27%). HF2/SC2/HF2 adiponectin significantly decreased in comparison with SC6 and was not different from HF6. In addition, a significant difference was found in adiponectin between SC2/HF2/SC2 and HF2/SC2/HF2 (−27%).

Comparing the same group at different ages, there was no differences in adiponectin levels between the SC groups. In relation to HF groups, there was statistical difference between HF2 and HF4 groups (+10%) and between HF2 and HF6 groups (+30%).

#### *b)* Serum Leptin

HF2, HF4 and HF6 leptin was significantly increased compared with the SC counterparts.

At 4 months: Leptin was also higher during HF and lower during SC feeding. In SC2/HF2, it significantly increased in comparison with SC4 (3,600%) and was not different from HF4. In addition, after the switch from HF to SC feeding, HF2/SC2 leptin levels significantly decreased in comparison with HF4 (−78%) and SC2/HF2 (−83%).

At 6 months: After three WCs, leptin decreased during SC and increased during HF feeding. SC2/HF2/SC2 leptin was not different from SC6 and was lower in comparison with the HF6 group (−89%). HF2/SC2/HF2 leptin significantly increased in comparison with SC6 (+709%) and was not different from the value found in HF6. In addition, a significant difference was found between SC2/HF2/SC2 and HF2/SC2/HF2 (+803%).

Comparing the same group at different ages, there was significant differences in serum leptin between the SC2 and SC6 groups (+236%) and between SC4 and SC6 groups (+2727%). In relation to HF groups, there was no difference between HF groups.

#### *c)* Serum Resistin

HF2, HF4 and HF6 resistin was significantly increased as compared with the SC counterparts.

At 4 months: SC2/HF2 resistin significantly increased in comparison with the SC4 group (+49%) and was not different from HF4. After the switch to SC, HF2/SC2 resistin significantly decreased in comparison with the SC2/HF2 group (−24%).

At 6 months: After three WCs, resistin decreased during SC and increased during HF feeding. SC2/HF2/SC2 resistin was not different from the SC6 group and was lower in comparison with the value found in the HF6 group (−39%). HF2/SC2/HF2 resistin significantly increased in comparison with SC6 (+40%) and was not different from the HF6 group. In addition, a significant difference was found between SC2/HF2/SC2 and HF2/SC2/HF2 (+65%).

Comparing the same group at different ages, there was no difference in serum resistin among the SC2, SC4 and SC6 groups and among the HF2, HF4 and HF6 groups.

#### *d)* Serum Interleukin-6 (IL-6)

HF2, HF4 and HF6 groups had IL-6 significantly higher compared with the SC counterparts.

At 4 months: SC2/HF2 IL-6 significantly increased in comparison with the SC4 group (+187%) and was not different from HF4. In addition, after the switch to SC, HF2/SC2 IL-6 significantly decreased in comparison with HF4 (−52%) and with SC2/HF2 (−49%).

At 6 months: After three WCs, the IL-6 decreased during the SC and increased during the HF feeding. SC2/HF2/SC2 IL-6 was higher in comparison with SC6 (+244%) and was not different from HF6. In addition, HF2/SC2/HF2 IL-6 significantly increased in comparison with SC6 (+339%) and was not different from HF6 and SC2/HF2/SC2.

Comparing the same group at different ages, there was no difference in serum IL-6 among the SC2, SC4 and SC6 groups and among the HF2, HF4 and HF6 groups.

#### *e)* Serum Tumor Necrosis Factor- (TNF)-alpha

TNF*-alpha* was significantly higher in HF2, HF4 and HF6 than in the SC counterparts.

At 4 months: TNF-alpha was higher during HF and lower during SC feeding. SC2/HF2 TNF-alpha significantly increased in comparison with SC4 (+50%) and was not different from HF4. In addition, after the switch to SC feeding, HF2/SC2 TNF-alpha was significantly lower than HF4 (−38%) and SC2/HF2 (−35%).

At 6 months: After three WCs, the TNF-alpha decreased during SC and increased during HF feeding. SC2/HF2/SC2 TNF-alpha was not different from SC6 and HF6. In addition, HF2/SC2/HF2 TNF-alpha significantly increased in comparison with SC6 (+47%) and was not different from HF6 and SC2/HF2/SC2.

Comparing the same group at different ages, there was no difference in TNF-alpha among the SC2, SC4 and SC6 groups and among the HF2, HF4 and HF6 groups.

#### *f)* Serum Monocyte Chemotactic Protein-1 – MCP-1

The MCP-1 of all of the groups at 2, 4 and 6 months of study showed no significant difference among the groups. Comparing the same group at different ages, there was no difference in MCP-1 among the SC2, SC4 and SC6 groups and among the HF2, HF4 and HF6 groups.

### Western blotting

#### *a)* Adiponectin expression (Figure 4)

**Figure 4:**
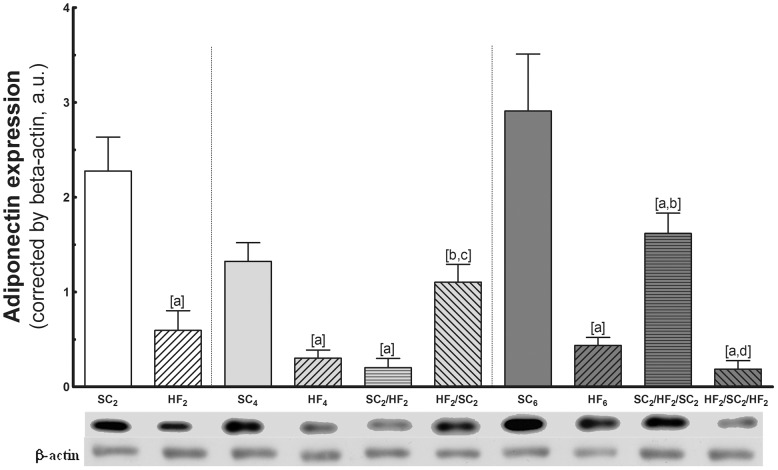
Adipose tissue expression of adiponectin. In the top, representative western blots with bands corresponding to groups, in order (n = 5 per group). Values are means with their standard errors shown by vertical bars. In the signaled cases (one-way ANOVA and post-hoc test of Bonferroni), *P<*0.05, when: [a] different from SC counterpart; [b] different from HF counterpart; [c] different from SC_2_/HF_2_; and [d] different from SC_2_/HF_2_/SC_2_.

HF2, HF4 and HF6 adiponectin expression was significantly lower as compared with SC counterparts.

At 4 months: Adiponectin was lower during HF and higher during SC feeding. SC2/HF2 adiponectin significantly decreased in comparison with SC4 (−85%) and was not different from HF4. In addition, after the switch to SC, HF2/SC2 adiponectin significantly increased in comparison with HF4 (+267%) and SC2/HF2 (+450%).

At 6 months: After three WC, adiponectin increased during SC and decreased during HF feeding. The adiponectin expression of SC2/HF2/SC2 was lower in comparison with SC6 (−44%) and was higher in comparison with the value found in HF6 (+268%). Adiponectin of HF2/SC2/HF2 significantly decreased in comparison with SC6 (−93%) and was not different from the value found in HF6. In addition, a significant difference was found between SC2/HF2/SC2 and HF2/SC2/HF2 (−88%).

Comparing the same group at different ages, there was difference only between SC4 and SC6 groups (+130%). In relation to HF groups, there was no statistical difference among the groups.

#### *b)* Leptin expression (Figure 5)

**Figure 5:**
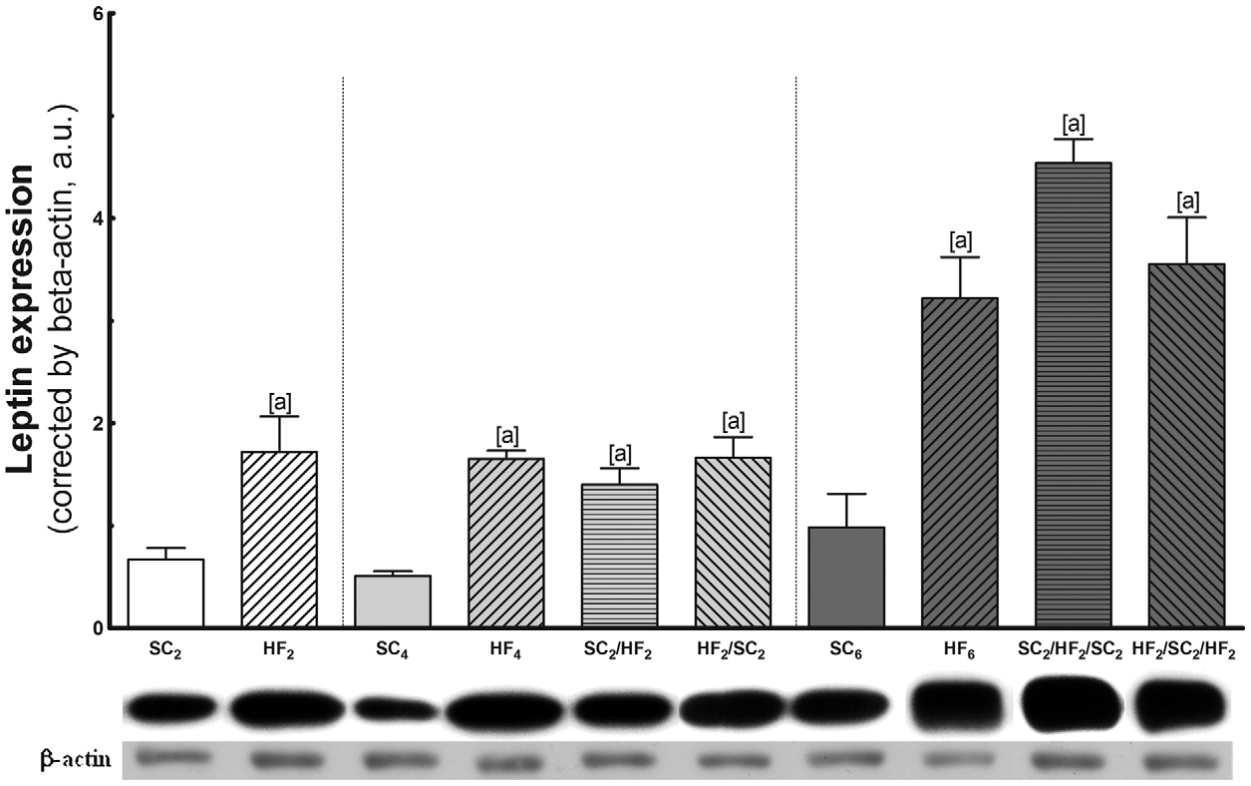
Adipose tissue expression of leptin. In the top, representative western blots with bands corresponding to groups, in order (n = 5 per group). Values are means with their standard error shown by vertical bars. In the signaled cases (one-way ANOVA and post-hoc test of Bonferroni), *P<*0.05, when: [a] different from SC counterpart; [b] different from HF counterpart; [c] different from SC_2_/HF_2_; and [d] different from SC_2_/HF_2_/SC_2_.

HF2, HF4 and HF6 leptin expression in intra-abdominal adipose tissue was significantly elevated as compared with SC counterparts.

At 4 months: Leptin expression in intra-abdominal adipose tissue was markedly increased in HF4, SC2/HF2 and HF2/SC2. Leptin in intra-abdominal adipose tissue remained constantly higher in these groups and did not fully normalize to the level of SC4.

At 6 months: After three WCs, leptin in intra-abdominal adipose tissue remained higher. In SC2/HF2/SC2, it was not different in comparison with HF6, but it was higher than the value found in SC6 (+363%). In addition, leptin in intra-abdominal adipose tissue of HF2/SC2/HF2 significantly increased in comparison with SC6 (+262%) and was not different from SC2/HF2/SC2.

Comparing the same group at different ages, there was no statistical difference among the SC groups. In relation to HF groups, there was statistical difference between HF2 and HF6 groups (+67%) and between HF4 and HF6 (+76%) groups.

#### *c)* Interleukine-6 (IL-6) expression (Figure 6)

**Figure 6:**
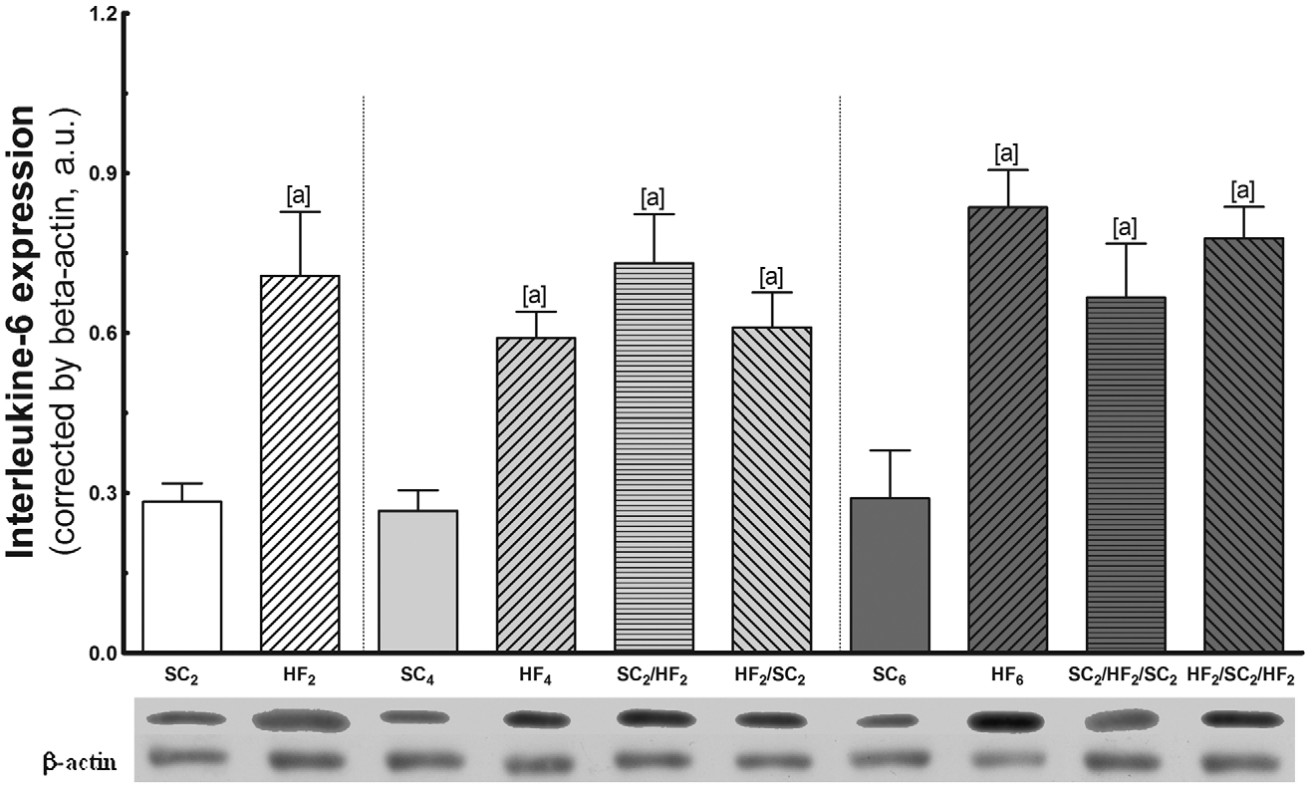
Adipose tissue expression of IL-6. At the top are representative western blots with bands that correspond to groups, in order (n = 5 per group). Values are means with their standard errors shown by vertical bars. In the signaled cases (one-way ANOVA and post-hoc test of Bonferroni), *P<*0.05, when: [a] different from SC counterpart; [b] different from HF counterpart; [c] different from SC_2_/HF_2_; and [d] different from SC_2_/HF_2_/SC_2_.

HF2, HF4 and HF6 IL-6 expression was significantly elevated as compared with the SC counterparts.

At 4 months: IL-6 expression was also markedly increased in SC2/HF2 and HF2/SC2. IL-6 remained constantly higher in these groups and did not fully normalize to the level of SC4.

At 6 months: After three WCs, the IL-6 remained higher. In SC2/HF2/SC2, it was not different in comparison with HF6, but it was higher than the value found in SC6 (+131%). In addition, IL-6 expression of HF2/SC2/HF2 significantly increased in comparison with SC6 (+169%) and was not different from SC2/HF2/SC2.

Comparing the same group at different ages, there was no difference in IL-6 among the SC2, SC4 and SC6 groups and among the HF2, HF4 and HF6 groups.

## Discussion

The present study clearly demonstrated that HF feeding and WC in mice were associated with significant alteration in the BM accompanied by a substantial modification of blood lipids, glucose homeostasis, adipokines, and the fat pads and adipocyte remodeling. Some of these parameters were recuperated when animals shift to SC. The SC2/HF2/SC2 group had significant reduction in BM, food intake, feed efficiency, serum cholesterol and triglycerides, fat pads, adiposity index, fat cell size, serum leptin, resistin, TNF-alpha and MCP-1. However, OGTT, insulin, adiponectin and IL-6 were not totally recuperated.

Moreover, it was demonstrated that the longer the feeding with HF happens, more severe is the adiposity in mice. Therefore, after three consecutive WC, the reduction of BM is less marked during the SC cycle, as well as the increase of BM is more prominent during HF cycle.

Present data showed that SC2/HF2 mice weighed as much as HF4 and the HF2/SC2/HF2 mice weighed as much as HF6. Thus, the mice that pass by two or three cycles of diet, after switching to SC diet, recuperated their BM. Humans and rodents under caloric restriction reduce their energy expenditure for energy conservation, a compensatory metabolic process responsible for the difficult to sustain weight loss indefinitely [14]. Moreover, BM loss causes greater than expected reduction in energy expenditure, which may persists during the weight-reduction, indicating that the decreased of energy expenditure is a metabolic defense against the weight loss [15]. Interestingly, frequent WCs are characterized by a reduction in the relative energy expenditure that explains why obese people with large fluctuations in BM have more prone to future weight gain [16], [17].

In the present study, both the food intake and feed efficiency were measured on mice submitted to WC and it was observed that the different experimental groups have taken approximately the same amount of the diet per gram of BM (no statistical difference). Therefore, as the HF diet has a greater energy than the SC diet there is a much higher feed efficiency in HF feeding mice in comparison to SC feeding mice. It has been argued that because the rate of carbohydrate and protein oxidation is determined by intake of these nutrients, whereas the rate of fat oxidation is determined largely by the gap between total energy expenditure and energy intake, obesity is the natural result of a diet rich in fat [18]. Likewise, a food restriction followed by a HF refeeding decreases oxygen utilization, leading to a same degree of adiposity as that caused by continuous fat feeding and the obese people remained obese as the ones maintained continuously on the high-energy diet [19]. The findings of the present study in mice are in agreement with those findings. Thus, the concomitant elevated FE with low energy expenditure can be a potential mechanism to predict weight gain and regain in mice.

We do not measure the spontaneous activity level in this study. However, it seems reasonable to assume that low activity can be another mechanism to gain weight in the HF feeding mice. Exercise training in rodents has an important impact on the body mass, as well as is able to treat the consequences of the obesity and HF diet [20].

In the present study, we found that the HF feeding and WC provoked a higher blood lipid levels and glucose intolerance. Switching to SC feeding has reduced the plasma lipid levels and had not a positive impact on the glucose tolerance or improved the serum insulin concentrations. Interestingly, the WC induced a fluctuation of serum TC and serum TG. The mechanism by which the HF diet increases plasma TG is thought to include increased liver TG production and VLDL secretion [21]. Additionally, serum cholesterol levels are closely correlated with BM and dietary fat intake [22]. The current study found high OGTT and high serum insulin in HF animals that were not recuperated by dietary shift to SC diet in agreement with literature reports [23]. The ingestion of HF diet for two months was deleterious to glucose tolerance and then the change to the SC diet may not have been enough to improve glucose tolerance and blood insulin levels, although there was a decrease in fat body mass and adiposity in this period.

In mice, the number of adipocytes stabilizes during the first four months of life, and later on [24], which is consistent with the present results. In SC2, SC4 and SC6 mice, the size and the density per area of adipocytes did not change during the experiment, while the adiposity index has increased. This increase in adiposity in the SC feeding animals throughout the experiment suggests a relationship with the ageing process in mice. It is well known that changes in body composition that are independent of BM changes are linked with ageing [25]. Therefore, changes in body fat and fat-free weight in subjects whose weight was stable during six years period reflect the expected loss in fat free weight and gain in body fat related to age [26].

The presence of macrophages in adipose tissue usually occurs after the rupture of adipocytes (death) because of a substantial cell enlargement resulting from the increased storage of triglycerides. The storage capacity of adipocytes depends on the expansion of the adipose tissue [27]. The larger the adipocyte is, the higher the probability of rupture. Hence, macrophages are recruited and aggregated in a crown-like structure to eliminate the cell contents that are released into the extracellular space [28]. In the present study, WC induced a fluctuation of fat pads, probably associated with adipocyte deaths. However, no areas free of adipocytes were observed, suggesting that the sites of adipocyte deaths were successfully remodeled to maintain the adipose tissue expansion. In addition, a striking feature of remodeled intra-abdominal adipose tissue was the prevalence of small adipocytes.

Although the adipocyte size has been not recuperated in the second cycle of diets, after switching HF to SC feeding, in the third cycle it was recuperated. This result also followed the results of the BM and adiposity. Moreover, the longer was the time of feeding HF diet, the more severe was the adiposity index of the animals. In HF2, HF4 and HF6 mice, the adipocytes size decreased with the time and showed higher density per unit area throughout the experiment, indicating that there was adipose tissue remodeling in these groups. Unlike what happens in humans, where the expansion of adipose tissue seems to be unlimited, in rodents, the increase in body-fat depots is limited. In an elegant work, it was demonstrated that mice fed HF diet for 20 weeks, the frequency of adipocyte death in adipose tissue increased from <0.1% at baseline to 16% at week 12. The adipocyte number began to decline at week 12. At week 16, adipocyte death reached approximately 80%. In addition, by the week 20, the number of adipocytes was restored with small adipocytes, coincident with reduced adipocyte death [29]. These results imply that the death of adipocytes and adipose tissue remodeling appears to be a hallmark of murine obesity.

The apoptosis of fat cells and the reestablishment of the adipocyte number with small adipocytes suggested that adipocyte death may be a prerequisite for transitions from hypertrophic to hyperplastic obesity in intra-abdominal adipose tissue in mice [30]. The adipocyte apoptosis is a key initial event that contributes to macrophage infiltration into adipose tissue, insulin resistance, and hepatic steatosis, which are associated with obesity in both mice and humans, suggesting that the inhibition of adipocyte apoptosis may be a new therapeutic strategy for the treatment of obesity-associated metabolic complications [31].

Intra-abdominal fat is that one closely associated with increased pathogenicity in obesity (such as inflammatory and metabolic complications), more than subcutaneous fat [29]. In addition, WC led to a more pronounced increase in intra-abdominal fat than in the subcutaneous fat pad. This result is relevant because the intra-abdominal adipose tissue secretes several adipokines, such as leptin, adiponectin, TNF-alpha and IL-6, which are involved in the modulation of inflammation [32]. Moreover, WC is associated with increased lipogenesis and the concomitant up-regulation of genes that are involved in fatty acid synthesis in rat white adipose tissue. These data revealed a potent pro-lipogenic effect of WC on adipose tissue metabolism [33].

Adiponectin is the most abundant circulating adipokine and has a potent anti-inflammatory effect. Circulating adiponectin concentration paradoxically decreases as obesity progresses [34]. Adiponectin levels are reduced in animal models of obesity and insulin resistance [35], and weight loss has been shown to increase adiponectin levels [36]. Moreover, circulating concentrations of adiponectin are lower in patients with diabetes, cardiovascular disease, and several malignancies [37]. In the present study, the HF diet and WC were associated with a substantial decrease in the adiponectin concentration in serum and adipose tissue expression, and this decrease was not reversible after WC. Interestingly, in the HF2/SC2 group (second cycle of diet), the adiponectin concentration in serum and its expression in adipose tissue were highest in both these compartments indicating a return of adiponectin levels to that of the SC4 mice, probably due to the shorter duration of the obesity. However, this increase did not occur in the SC2/HF2/SC2 group (third cycle of diet). Consistent with previous reports, the reduction of adiponectin is reportedly more pronounced if metabolic disturbances are present [38] and its reduced expression *in vivo* may be the result of cytokines (e.g. TNF-alpha, IL-6, or IL-8), which are released from surrounding cells, particularly pre-adipocytes and macrophages [39]. In this study, the decrease in adiponectin levels was better observed in the animals submitted to three cycles of diet, groups HF6, SC2/HF2/SC2 and HF2/SC2/HF2, which also showed high levels of TNF-alpha and IL-6.

Leptin is a hormone primarily synthesized and secreted by adipose tissue controlling the energy balance by binding to receptors in the hypothalamus, leading to a reduction in food intake and an elevation in the temperature and energy expenditure. Moreover, leptin is a pro-inflammatory molecule [40], and a higher circulating leptin concentration is associated with many autoimmune and inflammatory diseases [41]. Leptin expression seems to be higher in epididymal than in inguinal adipose tissue in rats [42]; however, weight loss and fasting are associated with reduced leptin levels, whereas weight gain is associated with an increase in the concentration of this hormone [43]. In addition, in abnormal situations that are characterized by chronic hyperleptinemia such as obesity, leptin may function pathophysiologically in the development of hypertension and possibly in direct renal, vascular, and cardiac damage [44]. In the present study, WC induced a fluctuation in the circulating levels of leptin. The HF feeding and WC were associated with an increased leptin expression in adipose tissue, which maintained the increase after WC. To begin to understand why leptin serum levels do not corresponded with the leptin expression in adipose tissue, we observed decreased serum leptin with SC feeding and an increased with HF feeding in mice. Leptin levels are also influenced by meal consumption and short-term swings in energy balance such as fasting or overfeeding [45], [46]. These dynamic changes in leptin serum levels due to WC in mice were accompanied by the fluctuation in feed efficiency, despite the animals had similar food consumption per grams. These different effects of the WC in serum leptin and leptin expression in adipose tissue are presumably due to extrinsic factors that also modulate the expression of leptin. In addition, the level of leptin expression indicates that leptin expression is modulated by other factors that act on adipose tissue. Insulin regulates the leptin expression directly in rats, regardless of its glucose-lowering effects, perhaps through a direct action of the insulin on the adipocyte [47]. Present data indicated high levels of leptin expression in HF mice and cycler mice in association with the high insulin levels in these animals.

Comparing the same group at different ages, there was significant differences in serum leptin among SC2, SC4 and SC6 groups, but not in HF groups. This is consistent with the adiposity index of the animals that progressed in SC mice throughout the experiment, while HF animals showed high adiposity index in all periods of observation. Therefore, the ageing seems be involved in the body adiposity and consequently with increased in leptin levels. In SC groups, the leptin expression in adipose tissue did not accompany age, probably due to the total amount of adipose tissue in the groups that was not increased as strongly as in HF groups. Moreover, in HF groups the adiposity was so greater in comparison with SC group that leptin expression in adipose tissue was maintained higher throughout the experiment.

Resistin, a cysteine-rich peptide that is secreted primarily from adipose tissue, is a unique signaling molecule that contributes to insulin resistance [48] and is strongly associated with inflammatory markers [49]. Resistin expression in vivo is specific to white adipose tissue and circulates in mouse serum, and its level is increased in both genetic and diet-induced obesity [50]. In the present study, WC induced a significant fluctuation of serum resistin, after WC serum resistin decreased during SC and increased during HF feeding. Serum resistin levels correlates with changes in BM and intra-abdominal fat [51]. Increased serum resistin levels and gene expression levels were observed in intra-abdominal fat depots in states of increased adiposity [52]. In addition, there is significant reduction in circulating resistin levels following moderate weight loss [53]. In the present study, WC induced a fluctuation of serum resistin associated with changes in the intra-abdominal and subcutaneous fat pads.

Adipose tissue is one of the major sources of IL-6, a pro-inflammatory cytokine that is elevated in states of obesity and insulin resistance [54]. The IL-6 serum levels increase during the intake of HF and decrease after the shift to a standard diet in mice [55], which is only partially in agreement with the present results. The IL-6 serum levels after two WCs were lowered by the shift to SC feeding and increased by the shift to HF feeding. However, after three WCs, IL-6 serum levels were not lowered by the shift to SC feeding and remained constantly high in these mice.

Another pro-inflammatory cytokine is TNF-alpha, a link between obesity, inflammation and diabetes [56]. TNF-alpha is expressed in and secreted by adipose tissue in association with the degree of adiposity and insulin resistance [57]. TNF-alpha levels increased in diet-induced obese mice compared to normal-weight mice [58] and might down-regulate adiponectin production [59]. However, adiponectin reduces the production and activity of TNF-alpha [60]. In the present study, WC induced a significant fluctuation of serum TNF-alpha with a decrease during SC and an increase during HF feeding.

In general, the literature reports the MCP-1 levels increases in obesity. MCP-1 is produced predominantly by macrophages and endothelial cells and is a potent chemokine that is elevated in obesity and the increase of MCP-1 expression in adipose tissue contributes to macrophage infiltration and insulin resistance that is associated with obesity [61]. However, in the present study, HF feeding or WC did not change the serum levels of MCP-1 during the weight gain or weight loss, which agrees with a previous study of mice fed HF diet from our group [62]. This apparent discrepancy should be considered carefully because, in part, it could be explained due to differences in the species of animals studied (rats *versus* mice, for example), as well as the time duration of the study, or the diet composition. The level of MCP-1 is increased in mice fed HF diet for a short period of time [63], but the level of MCP-1 in longstanding obesity, is decreased probably as a manifestation of reduced pro-inflammatory activity of immune cells in the adipose tissue [64]. Therefore, present findings of no changes in the serum levels of MCP-1 during the gain and loss of BM could reflect the adipocyte dysregulation in WC mice.

In conclusion, the results of the present study showed that both the HF diet and WC are relevant to BM evolution and fat pad remodeling in mice, with repercussion in blood lipids, homeostasis of glucose-insulin and adipokine levels. The simple reduction of the BM during a WC is not able to recover the high levels of adipokines in the serum and adipose tissue as well as the pro-inflammatory cytokines enhanced during a cycle of HF diet. These findings are significant because a milieu with altered adipokines in association with WC potentially aggravates the chronic inflammation attributed to dysregulated production and release of adipokines in mice.

## References

[1] Olefsky JM, Glass CK (2010). Macrophages, inflammation, and insulin resistance.. Annu Rev Physiol.

[2] Delbue S, Guerini FR, Mancuso R, Caputo D, Mazziotti R (2007). JC virus viremia in interferon-beta -treated and untreated Italian multiple sclerosis patients and healthy controls.. J Neurovirol.

[3] Gruson E, Montaye M, Kee F, Wagner A, Bingham A (2010). Anthropometric assessment of abdominal obesity and coronary heart disease risk in men: the PRIME study.. Heart.

[4] Casanueva FF, Moreno B, Rodriguez-Azeredo R, Massien C, Conthe P (2010). Relationship of abdominal obesity with cardiovascular disease, diabetes and hyperlipidaemia in Spain.. Clin Endocrinol (Oxf).

[5] Flint AJ, Hu FB, Glynn RJ, Caspard H, Manson JE (2010). Excess weight and the risk of incident coronary heart disease among men and women.. Obesity (Silver Spring).

[6] Blackburn GL, Wilson GT, Kanders BS, Stein LJ, Lavin PT (1989). Weight cycling: the experience of human dieters.. Am J Clin Nutr.

[7] Strohacker K, McFarlin BK (2010). Influence of obesity, physical inactivity, and weight cycling on chronic inflammation.. Front Biosci (Elite Ed).

[8] Taing KY, Ardern CI, Kuk JL (2011). Effect of the Timing of Weight Cycling During Adulthood on Mortality Risk in Overweight and Obese Postmenopausal Women.

[9] Field AE, Malspeis S, Willett WC (2009). Weight cycling and mortality among middle-aged or older women.. Arch Intern Med.

[10] Reeves PG, Rossow KL, Lindlauf J (1993). Development and testing of the AIN-93 purified diets for rodents: results on growth, kidney calcification and bone mineralization in rats and mice.. J Nutr.

[11] Tschanz SA, Burri PH, Weibel ER (2011). A simple tool for stereological assessment of digital images: the STEPanizer.. J Microsc.

[12] Gundersen HJ (1977). Notes on the estimation of the numerical density of arbitrary profiles: the edge effect.. J Microsc.

[13] Jolicoeur P, Heusner AA (1986). Log-normal variation belts for growth curves.. Biometrics.

[14] Leibel RL, Rosenbaum M, Hirsch J (1995). Changes in energy expenditure resulting from altered body weight.. N Engl J Med.

[15] Astrup A, Gotzsche PC, van de Werken K, Ranneries C, Toubro S (1999). Meta-analysis of resting metabolic rate in formerly obese subjects.. Am J Clin Nutr.

[16] Strychar I, Lavoie ME, Messier L, Karelis AD, Doucet E (2009). Anthropometric, metabolic, psychosocial, and dietary characteristics of overweight/obese postmenopausal women with a history of weight cycling: a MONET (Montreal Ottawa New Emerging Team) study.. J Am Diet Assoc.

[17] Korkeila M, Rissanen A, Kaprio J, Sorensen TI, Koskenvuo M (1999). Weight-loss attempts and risk of major weight gain: a prospective study in Finnish adults.. Am J Clin Nutr.

[18] Flatt JP (1987). Dietary fat, carbohydrate balance, and weight maintenance: effects of exercise.. Am J Clin Nutr.

[19] Rozen R, Brigant L, Apfelbaum M (1994). Effects of cycles of food restriction followed by ad libitum refeeding on body composition and energy expenditure in obese rats.. The American journal of clinical nutrition.

[20] Marques CM, Motta VF, Torres TS, Aguila MB, Mandarim-de-Lacerda CA (2010). Beneficial effects of exercise training (treadmill) on insulin resistance and nonalcoholic fatty liver disease in high-fat fed C57BL/6 mice.. Braz J Med Biol Res.

[21] Olefsky JM, Farquhar JW, Reaven GM (1974). Reappraisal of the role of insulin in hypertriglyceridemia.. Am J Med.

[22] Giesen K, Plum L, Kluge R, Ortlepp J, Joost HG (2003). Diet-dependent obesity and hypercholesterolemia in the New Zealand obese mouse: identification of a quantitative trait locus for elevated serum cholesterol on the distal mouse chromosome 5.. Biochem Biophys Res Commun.

[23] Lee IS, Shin G, Choue R (2010). Shifts in diet from high fat to high carbohydrate improved levels of adipokines and pro-inflammatory cytokines in mice fed a high-fat diet.. Endocrine journal.

[24] Lemonnier D (1972). Effect of age, sex, and sites on the cellularity of the adipose tissue in mice and rats rendered obese by a high-fat diet.. J Clin Invest.

[25] Forbes GB (2000). Body fat content influences the body composition response to nutrition and exercise.. Ann N Y Acad Sci.

[26] Heitmann BL, Garby L (2002). Composition (lean and fat tissue) of weight changes in adult Danes.. Am J Clin Nutr.

[27] Smith J, Al-Amri M, Dorairaj P, Sniderman A (2006). The adipocyte life cycle hypothesis.. Clin Sci (Lond).

[28] Cinti S, Mitchell G, Barbatelli G, Murano I, Ceresi E (2005). Adipocyte death defines macrophage localization and function in adipose tissue of obese mice and humans.. J Lipid Res.

[29] Strissel KJ, Stancheva Z, Miyoshi H, Perfield JW 2nd, DeFuria J, et al (2007). Adipocyte death, adipose tissue remodeling, and obesity complications.. Diabetes.

[30] Faust IM, Johnson PR, Stern JS, Hirsch J (1978). Diet-induced adipocyte number increase in adult rats: a new model of obesity.. Am J Physiol.

[31] Alkhouri N, Gornicka A, Berk MP, Thapaliya S, Dixon LJ (2010). Adipocyte apoptosis, a link between obesity, insulin resistance, and hepatic steatosis.. J Biol Chem.

[32] Ahima RS (2006). Adipose tissue as an endocrine organ.. Obesity (Silver Spring).

[33] Kochan Z, Karbowska J, Swierczynski J (2006). The effects of weight cycling on serum leptin levels and lipogenic enzyme activities in adipose tissue.. J Physiol Pharmacol.

[34] Peake PW, Kriketos AD, Campbell LV, Shen Y, Charlesworth JA (2005). The metabolism of isoforms of human adiponectin: studies in human subjects and in experimental animals.. Eur J Endocrinol.

[35] Fumeron F, Aubert R, Siddiq A, Betoulle D, Pean F (2004). Adiponectin gene polymorphisms and adiponectin levels are independently associated with the development of hyperglycemia during a 3-year period: the epidemiologic data on the insulin resistance syndrome prospective study.. Diabetes.

[36] Moreno-Navarrete JM, Catalan V, Ortega F, Gomez-Ambrosi J, Ricart W (2010). Circulating omentin concentration increases after weight loss.. Nutr Metab (Lond).

[37] Ziemke F, Mantzoros CS (2010). Adiponectin in insulin resistance: lessons from translational research.. Am J Clin Nutr.

[38] Xydakis AM, Case CC, Jones PH, Hoogeveen RC, Liu MY (2004). Adiponectin, inflammation, and the expression of the metabolic syndrome in obese individuals: the impact of rapid weight loss through caloric restriction.. J Clin Endocrinol Metab.

[39] Skurk T, Alberti-Huber C, Herder C, Hauner H (2007). Relationship between adipocyte size and adipokine expression and secretion.. J Clin Endocrinol Metab.

[40] Tsochatzis E, Papatheodoridis GV, Archimandritis AJ (2006). The evolving role of leptin and adiponectin in chronic liver diseases.. Am J Gastroenterol.

[41] Fantuzzi G, Faggioni R (2000). Leptin in the regulation of immunity, inflammation, and hematopoiesis.. J Leukoc Biol.

[42] Zhang Y, Guo KY, Diaz PA, Heo M, Leibel RL (2002). Determinants of leptin gene expression in fat depots of lean mice.. Am J Physiol Regul Integr Comp Physiol.

[43] Rosenbaum M, Nicolson M, Hirsch J, Murphy E, Chu F (1997). Effects of weight change on plasma leptin concentrations and energy expenditure.. J Clin Endocrinol Metab.

[44] Kshatriya S, Liu K, Salah A, Szombathy T, Freeman RH (2011). Obesity hypertension: the regulatory role of leptin.. Int J Hypertens.

[45] Shi H, Strader AD, Woods SC, Seeley RJ (2007). Sexually dimorphic responses to fat loss after caloric restriction or surgical lipectomy.. Am J Physiol Endocrinol Metab.

[46] Maffei M, Halaas J, Ravussin E, Pratley RE, Lee GH (1995). Leptin levels in human and rodent: measurement of plasma leptin and ob RNA in obese and weight-reduced subjects.. Nat Med.

[47] Saladin R, De Vos P, Guerre-Millo M, Leturque A, Girard J (1995). Transient increase in obese gene expression after food intake or insulin administration.. Nature.

[48] Steppan CM, Lazar MA (2002). Resistin and obesity-associated insulin resistance.. Trends Endocrinol Metab.

[49] Qi Q, Wang J, Li H, Yu Z, Ye X (2008). Associations of resistin with inflammatory and fibrinolytic markers, insulin resistance, and metabolic syndrome in middle-aged and older Chinese.. Eur J Endocrinol.

[50] Steppan CM, Bailey ST, Bhat S, Brown EJ, Banerjee RR (2001). The hormone resistin links obesity to diabetes.. Nature.

[51] Azuma K, Katsukawa F, Oguchi S, Murata M, Yamazaki H (2003). Correlation between serum resistin level and adiposity in obese individuals.. Obes Res.

[52] McTernan PG, McTernan CL, Chetty R, Jenner K, Fisher FM (2002). Increased resistin gene and protein expression in human abdominal adipose tissue.. J Clin Endocrinol Metab.

[53] Valsamakis G, McTernan PG, Chetty R, Al Daghri N, Field A (2004). Modest weight loss and reduction in waist circumference after medical treatment are associated with favorable changes in serum adipocytokines.. Metabolism.

[54] Nieto-Vazquez I, Fernandez-Veledo S, de Alvaro C, Lorenzo M (2008). Dual role of interleukin-6 in regulating insulin sensitivity in murine skeletal muscle.. Diabetes.

[55] Lee IS, Shin G, Choue R (2010). Shifts in diet from high fat to high carbohydrate improved levels of adipokines and pro-inflammatory cytokines in mice fed a high-fat diet.. Endocr J.

[56] Hotamisligil GS, Arner P, Caro JF, Atkinson RL, Spiegelman BM (1995). Increased adipose tissue expression of tumor necrosis factor-alpha in human obesity and insulin resistance.. J Clin Invest.

[57] Tzanavari T, Giannogonas P, Karalis KP (2010). TNF-alpha and obesity.. Curr Dir Autoimmun.

[58] Morin CL, Eckel RH, Marcel T, Pagliassotti MJ (1997). High fat diets elevate adipose tissue-derived tumor necrosis factor-alpha activity.. Endocrinology.

[59] Chandran M, Phillips SA, Ciaraldi T, Henry RR (2003). Adiponectin: more than just another fat cell hormone?. Diabetes Care.

[60] Masaki T, Chiba S, Tatsukawa H, Yasuda T, Noguchi H (2004). Adiponectin protects LPS-induced liver injury through modulation of TNF-alpha in KK-Ay obese mice.. Hepatology.

[61] Ohman MK, Eitzman DT (2009). Targeting MCP-1 to reduce vascular complications of obesity.. Recent Pat Cardiovasc Drug Discov.

[62] Catta-Preta M, Martins MA, Cunha Brunini TM, Mendes-Ribeiro AC, Mandarim-de-Lacerda CA (2012). Modulation of cytokines, resistin, and distribution of adipose tissue in C57BL/6 mice by different high-fat diets.. Nutrition.

[63] Ito A, Suganami T, Miyamoto Y, Yoshimasa Y, Takeya M (2007). Role of MAPK phosphatase-1 in the induction of monocyte chemoattractant protein-1 during the course of adipocyte hypertrophy.. J Biol Chem.

[64] Altintas MM, Rossetti MA, Nayer B, Puig A, Zagallo P (2011). Apoptosis, mastocytosis, and diminished adipocytokine gene expression accompany reduced epididymal fat mass in long-standing diet-induced obese mice.. Lipids Health Dis.

